# Effect of gut microbiota on colorectal cancer progression and treatment

**DOI:** 10.15537/smj.2022.43.12.20220367

**Published:** 2022-12

**Authors:** Glowi A. Alasiri

**Affiliations:** *From the Department of Biochemistry, College of Medicine, Al Imam Mohammad Ibn Saud Islamic University, Riyadh, Kingdom of Saudi Arabia.*

**Keywords:** gut microbiota, colorectal cancer, metabolites, treatment

## Abstract

Microbiota is a collection of bacteria, archaea, eukaryotes, bacteriophages, viruses, and fungi that cover human body surfaces and cavities. They characterize inside the body due to several factors such as diet, nutrition, xenobiotic substances, and microbial infections. Several studies have shown that gut microbiota can induce resistance against pathogens and regulate the immune system. In addition, their disruption is associated with several physiological and biochemical disorders, including inflammatory bowel disease (IBD), obesity, autoimmune diseases such as diabetes, hypertension, colon cancer, and cardiovascular disease. Colorectal cancer (CRC) is the third-deadliest cancer worldwide, accounting for approximately 900,000 deaths per year globally. Gut microbiota has been heavily linked to CRC incidence and prevention via bacterial metabolites, invasion, translocation, host’s defense modulations, and bacterial-immune system interactions. In addition, it can influence the metabolism of chemical compounds such as drugs and xenobiotics to manipulate the treatment response in CRC patients.

**M**icrobiota is a collection of bacteria, archaea, bacteriophages, eukaryotes, viruses, and fungi that cover human body surfaces and cavities.^1^ The gut microbiome is considered a health indicator that is sensitive to environmental, dietary, and host’s genetic factors.^2^ Gut microbiota can induce resistance to pathogens and regulate the immune system.^3^ As a result, disruption of gut microbiota is associated with several physiological and biochemical disorders, including inflammatory bowel disease (IBD), obesity, autoimmune diseases such as diabetes, hypertension, colorectal cancer (CRC), and cardiovascular disease.^4^ In a person’s early life, gut microbiota develops through breastfeeding during their infancy, and it matures with age and exposure to environmental factors.^5^ However, in late childhood, microbiota composition and maturation stabilize and the composition begins to experience imbalances in late adulthood.^5^ The formation, diversity, and function of gut microbiota differ based on gender, ethnicity, nutrition, age, and health conditions of the host.^6^ This review article discusses in depth the role of gut microbiota and its functions to explore the link between development of CRC in patients and their responses to treatment.

## Mechanisms that lead to imbalance of gut microbiota

**Figure 1:**
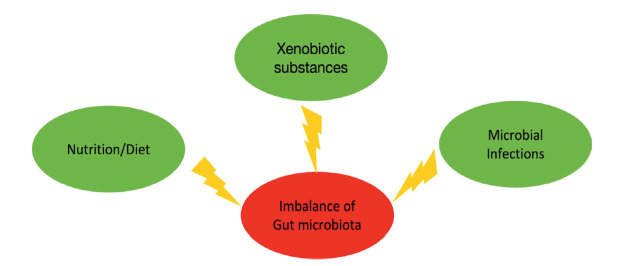
- Imbalance of gut microbiota via diet, infections, nutrition, and xenobiotic.

Gut microbiota composition is characterized by several factors such as diet, nutrition, xenobiotic substances, and microbial infections.^7^ Perturbations of these factors might decrease the diversity of gut microbiota, leading to depletion of health-related microbiome and an increase in microbial pathogens.^7^ These perturbations could last for short durations in an acute state, affecting the gut microbiota compositions.^8^ However, a chronic state of such perturbations will induce alterations in the function and composition of gut microbiota that may result in persistence of such perturbations (Figure 1).^8^

## Nutrients/diet

Gut microbiota mainly derives its nutrition from the fermentation of carbohydrates, lipids, and amino acids.^9^ For carbohydrates, microbiota colonies including *Bacteroides*, *Roseburia*, *Bifidobacterium*, *Faecalibacterium*, and *Enterobacteriaceae*, target indigestible oligosaccharides and carbohydrates, resulting in the synthesis of short-chain fatty acids (SCFAs).^10^ These SCFAs regulate hosts’ energy generation and remove toxins through carbohydrate metabolism. For instance, tyrosine/pancreatic peptide YY3-36, which regulates the hosts’ energy balance, interacts with SCFAs.^11^ Additionally, the SCFA component butyrate reduces the buildup of harmful byproducts such D-lactate.12 This process is carried out by *Bacteroides* such as *Bacteroides thetaiotaomicron* (*B. thetaiotaomicron*), which produce enzymes including glycosyltransferases, glycoside hydrolases, and polysaccharide lyases.^12^ Furthermore, carbohydrate fermentation and bacterial metabolism lead to the production of harmful elements such as oxalates. As a result, other microbiota species including Oxalobacter formigenes, Lactobacillus species, and Bifidobacterium, eliminate this product, preventing kidney stones.^13^

In lipid metabolism, gut microbiota can regulate triglyceride breakdown by preventing inhibition of lipoprotein lipase activity in the adipocyte.^14^ In addition, B. thetaiotaomicron has been shown to induce the colipase activity required for lipid digestion.^15^ Additionally, human proteinases found in microbial proteinases and peptidases enable gut microbiota to digest proteins. Amino acid transporters are part of the bacterial cell wall and aid in the absorption of amino acids, which are then converted into small signaling molecules and antimicrobial peptides (bacteriocins).^16^ For example, L-histidine and glutamate are converted to histamine and γ-aminobutyric acid (GABA) through the regulation of histamine and glutamate decarboxylases.^17^ However, protein metabolism can be harmful to human due to the production of branched-chain amino acids, various phenolics, and other metabolites that are toxic to the host.18 In addition, the fermentation of amino acids produces compounds that are associated with gut diseases such as IBD and CRC.^18^

**Figure 2:**
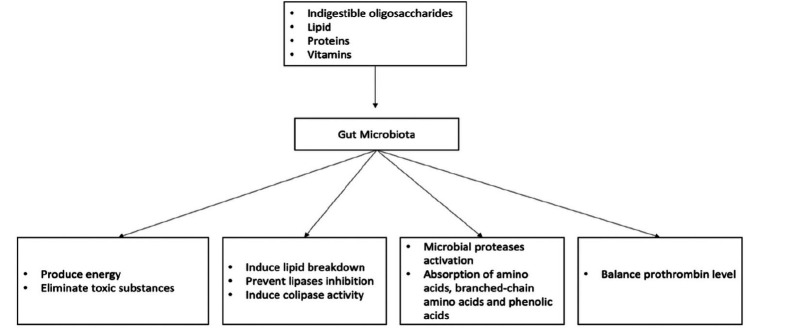
- Effects of gut microbiota on nutrition.

Previous studies on gut microbiota have demonstrated that it can help in the production of vitamins like vitamin K and a number of vitamin-B components such as biotin, cobalamin, folic acid, nicotinic acid, pantothenic acid, pyridoxine, riboflavin, and thiamine.^19^ For instance, thrombin levels associated with hemorrhage formation were lower in germ- and vitamin K-free animals than in conventional mice with average prothrombin levels and clotting activity.^20^ An overview of the impact of gut microbiota on nutrition is shown in Figure 2.

Fruits and vegetables are sources of polyphenols such as phenolic acids, flavonoids, stilbenes, lignans, and secoiridoids, which are needed by the body for their bioactivity roles.^21^ In general, polyphenols are mainly absorbed by colonic microbiota due to their poor absorption in the small intestine.^22^ Polyphenols emerge as glycosidase conjugated to monosaccharides such as glucose, galactose, rhamnose, and rutinose.^9^ These glycosidases are hydrolyzed by microbiota species such as *Bacteroides distasonis* (*B. distasonis*), *Bacteroides uniformis* (*B. uniformis*), *Bacteroides ovatus* (*B. ovatus*), *Enterococcus casseliflavus* (*E. casseliflavus*), *Eubacterium cellulosolvens* (*E. cellulosolvens*), *Lachnospiraceae CG19-1*, and *Eubacterium ramulus* (*E. ramulus*).^9^ Polyphenols are inactive until the removal of sugar by microbiota, which induces their activity concerning the structure of polyphenols and richness of microbiota in the colon.^22^ An example of the biotransformation of polyphenols is the derivation of aglycone and equol from inactive isoflavones as antiandrogenic and hypolipidemic effects.^23^

## Inflammation due to specific nutrients/diet

The development of gut microbiota in the human body starts in early infancy through breastfeeding and is related a mother’s diet during her pregnancy.^24^ Previous studies have shown that gut microbiota differs between breastfed and formula-fed infants.^25^ Studies showed that breast milk was composed of bioactive compounds that supported nutrient digestion and absorption, immune protection, and antimicrobial defenses.^26^ Also, gut microbiota such as *Bifidobacterium species* cause carbohydrate fermentation of SCFAs and butyrate, and regulate the immune system.^27^ In addition, different groups of bacterial colonies such as the *Bacteroides* and *Clostridium* *species*, dominantly found in formula-fed infants compared to breastfed infants, suggests the nutrition selectivity of gut microbiota colonization.^28^ Furthermore, a recent study showed that breastfed infants have less microbiota diversity than formula-fed infants, which further supports the notion that diet shapes the development of gut microbiota from an early age.^25^

Another diet-related perturbation of gut microbiota is the type of nutrients consumed. Gut microbiota in a healthy state requires a diet rich in fruits, vegetables, and fiber that produce indigestible molecules required by metabolizing organisms such as the *Firmicutes strain*, *Ruminococcus bromii*, *Roseburia*, and *Eubacterium rectale*.^29^ The colonization of bile-tolerant bacteria in meat-based diets and *Firmicutes* phylum in plant-based diets has also been shown in studies to be more pronounced in those consuming meat-based diets than plant-based diets.^30^

Seasonal and geographic variables can also affect the makeup of the gut microbiota. Children in Europe have a diet rich in protein, sugar, and carbohydrate and poor in fiber, which is related to dominance of *Bacteroides* in their diets. On the contrary, children in Africa have an agricultural diet linked to the dominance of *Prevotella*.^31^ Additionally, environment can impact the makeup and diversity of gut microbiota. For instance, an investigation on the impact of seasons on gut microbiota in the Ukrainian population revealed that *Actinobacteria* were dominant and *Bacteroidetes* were reduced during the summer whereas *Firmicutes* were more stable and unaffected by seasonal changes.^32^

Polyphenols are present in a broad range of plant foods and interact with epithelium cells to modulate the composition of gut microbiota.^33^ Metabolites produced from polyphenols mainly induce *Lactobacillus* strains that inhibit the pathogenic action of some bacteria such as *Salmonella* and *Helicobacter pylori* (*H. pylori*) species.^33^ Gut microbiota such as *B. distasonis*, *B. uniformis*, *B. ovatus*, *E. casseliflavus*, and *E. ramulus* can degrade quercetin and polyphenol metabolites with flavanol.^9^ However, flavanol is an aglycone that has been shown to have an inhibitory effect on *Staphylococcus aureus* and *H. pylori*, which are vancomycin intermediates.^34^

Vitamins and minerals play an essential role in regulating the symbiotic relationship between hosts and gut microbiota.^35^ For example, vitamins K and B are produced by gut microbiota and shared among species through cross-feeding interactions.^36^ Additionally, minerals act as cofactors required by hosts and bacteria, and play an essential role in the growth selectivity of gut microbiota.^35^ For instance, an elevated level of iron is related to the growth of pathogenic bacteria.^37^

To this end, a diet that provides balanced nutrition maintains diversity and composition of gut microbiota, while a diet high in fat or protein leads to a decrease in bacterial composition and an increase in the host’s susceptibility to pathogenic infection. Therefore, the composition of a host’s gut microbiota is essential for recommending them with appropriate nutrition and diet.

## Gut microbiota species and metabolites associated with CRC or chronic inflammation

Gut microbiota imbalance or dysbiosis is associated with several diseases, including IBD, CRC, obesity, diabetes mellitus, and autism spectrum disorders. The main gut microbiota species in the human intestine are *Firmicutes*, *Bacteroidetes*, *Actinobacteri*, and *Proteobacteria*.^38^ Perturbation of gut microbiota induces metabolites that create chronic inflammation, leading to the production of carcinogenic agents. Factors such as nutrition, antibiotics, and environment, affect gut microbiota composition and decrease their diversity; this is associated with the occurrence of several diseases.

Colorectal cancer is the third-deadliest cancer in the world, accounting for approximately 900,000 deaths each year globally.^39^ Several studies have connected CRC to factors such as nutrition and physical activity, which also effect modulation of gut microbiota. Indeed, the gut microbiome manipulates CRC development directly and indirectly through bacterial metabolites, invasion, translocation, host’s defense modulations, and bacterial-immune system interactions.^40^ Therefore, in this section, the relation of gut microbiota species and their metabolites to CRC will be discussed (Tables 1 & 2).

**Table 1:** - Bacteria associated with colorectal cancer.

Bacteria	Association with colorectal cancer
*Fusobacterium nucleatum*	- Stages, recurrence, and patients’ low survival rates - Prevalence of a molecular feature such as mutation of BRAF, hypermutation with microsatellite instability and metastases - Serrated pathway manipulation - Binds to the E-cadherins’s extracellular domain and induces cancer cell proliferation - Promotes autophagy by upregulating CARD3 - Regulates microRNA to induce chemotherapy resistance - Inhibits T-cell infiltration and stimulates myeloid-derived immunity
*Peptostreptococcus anaerobius*	- Induces TLR2/4 that causes ROS activation - Promotes cholesterol synthesis - Induces cell proliferation
*Peptostreptococcus stomatis*	- Induces hypoxia - Produces CMS-1
*Prevotella intermedia*	Mutates P53 in pancreatic cancer
*Parvimonas micra*	Interrupts NOD2 that is involved in chemotherapy resistance and cancer progression
*Bacteroides fragilis*	- Regulates STAT3 and NF-kB that modulate pro-inflammatory molecules such as cytokine IL-17 - Interrupts E-cadherin and DNA damage - Induces COX-2, causing upregulation of PGE2 and promotion of inflammation and cell proliferation
*Streptococcus gallolyticus*	Induces pro-inflammatory markers such as NF-κB and IL-8
*Escherichia coli*	Induces chromosomal appearance

BRAF: B-Raf Proto-Oncogene, Serine/Threonine Kinase, CARD3: caspase activation and recruitment domain 3, TLR: toll-like receptor, ROS: reactive oxygen species, CMS-1: consensus molecular subtype-1, P53: tumor protein p53, NOD2: nucleotide-binding oligomerization domain 2, STAT3: Signal Transducer And Activator Of Transcription 3, NF-kB: nuclear factor kappa B, IL: interleukin, COX-2: cyclooxygenase-2, PGE2: prostaglandin E2

**Table 2:** - Bacterial metabolites and colorectal cancer.

Bacterial metabolites	Examples	Effect on CRC
SCFAs	Butyrate, propionate, and acetate	- Immunomodulation via regulation of AhR - Rebalance gut microbiota diversity and composition - Inhibit NF-kB and activate apoptosis - Antitumor effect via modulation of Tc17 cells and CTLs - Inhibit ERK1/2, causing tumor growth disruption - Induce ROS activity and decrease glucose oxidation - Induce HDAC
Bile acid	Primary and secondary bile acids	- Bind to FXR as CRC inhibitor - Promote cancer initiation by upregulating IL-8, ERK1/2, and inhibiting STAT3 phosphorylation - Activate MAPK pathway via upregulation of uPAR and calcium signaling
Lactate		- Creates an acidification environment - Stimulates angiogenic response to oxygen transfer, glucose delivery and nutrition delivery that promote CRC invasion, proliferation, and migration
Succinate		- Inhibits CRC proliferation and induces CRC metastasis via SUCNR1 signaling
Protein-derived metabolites	HO-PAA, PAA, phenol (produced from tyrosine), acetaldehyde, H2S, and NOCs	- Hydrogen sulfide inhibits the anti-inflammatory outcome in CRC cell lines by activating NF-kB pathway signaling - NOCs contribute to K-ras mutation, which drives CRC proliferation

SCFAs: short-chain fatty acids, HO-PAA: 4-hydroxyphenylacetic acid, PAA: phenylacetic acid, H2S: hydrogen sulfide, NOCs: N-nitroso compounds, AhR: aryl hydrocarbon receptor, NF-kB: nuclear factor kappa B, Tc17: IL-17-secreting CD8 T cells), CTLs: cytotoxic T lymphocytes, ERK1/2: protein kinases 1 and 2, ROS: reactive oxygen species, HDAC: histone deacetylases, FXR: farnesoid X receptor, CRC: colorectal cancer, IL: interleukin, STAT3: Signal Transducer and Activator of Transcription 3, MAPK: mitogen-activated protein kinase, uPAR: urokinase plasminogen activator, SUCNR1: succinate receptor 1, K-ras: Kirsten rat sarcoma viral oncogene homolog

## Bacteria associated with CRC

Several studies have found elevated F. nucleatum DNA and RNA sequences in tumor patients compared to non-tumor patients, including in CRC patients.^41^ In addition, *F. nucleatum* was further linked to the stages of CRC, recurrence, and patients’ low survival rates.^42^ Moreover, studies have shown an elevation of *F. nucleatum* prevalence with a molecular feature of CRC, such as the mutation of B-Raf Proto-Oncogene, Serine/Threonine Kinase, hypermutation with microsatellite instability, and metastases.^43^ In addition, the serrated pathway was discovered to be manipulated by *F. nucleatum* in CRC.^41^ Furthermore, FadA (a virulence factor of *F. nucleatum*) can bind to the extracellular domain of E-cadherins and induce cancer cell proliferation.^41^ In addition, CRC patients with metastasis have an abundant level of *F. nucleatum*, which has been demonstrated to promote autophagy through the upregulation of caspase activation and recruitment domain 3 expression.^44^ Using metformin to treat *Adenomatous polyposis coli* (APC) mice showed that *F. nucleatum*-induced tumorigenicity in APCMin/+ mice diminished, suggesting that *F. nucleatum* is the driving factor of CRC.^45^

Consistent with its role in CRC, *F. nucleatum* has been linked to the inhibition of T-cell infiltration and stimulation of myeloid-derived immunity, which reflects *F. nucleatum* in the regulation of immune responses in CRC patients.^46^ Furthermore, *F. nucleatum* modulates CRC by suppressing antitumor responses as it enables the expansion of scurfin (FOXP3) non-Treg cells.^47^ Macrophage activation in CRC patients results from the elevation of miRNA-21, which induces pro-inflammatory interleukin-10 (IL-10) and prostaglandin E2, leading to T-cell suppression of cell antitumor functions.^48^ Additionally, fusobacterial protein (Fap2) binds to the T cell immunoreceptor with immunoglobulin and immunoreceptor tyrosine-based inhibitory motif domains inhibitory receptors at natural killer cells, leading to immunosuppression in CRC patients.^49^ In addition, Fap2 is also involved in inducing cytokines IL-8 and The chemokine (C-X-C motif) ligand one that promote CRC migration and progression.^50^

*Fusobacterium nucleatum* (*F. nucleatum*) induces chemotherapeutic resistance in CRC patients through the regulation of innate immune responses. Innate immune receptors such as toll-like receptor 4 (TLR4), myeloid differentiation primary response 88, and specific miRNA such as miR18a and miR4082, are activated by *F. nucleatum* to trigger autophagy and induce chemotherapy-resistance.^51^ In addition, *F. nucleatum* associates with TLR4/nuclear factor kappa B (NF-kB) to promote Baculoviral IAP Repeat Containing 3 expression and obstruct apoptosis.^52^

*Peptostreptococcus* species is one of the gut microbiota that is enriched in CRC patients.^53^ Two types of *Peptostreptococcus* species are connected to CRC; *P. stomatis* and *P. anaerobius*.^54^

High amounts of *P. anaerobius* are present in CRC patients, indicating a connection between them.^55^ Further investigation has demonstrated that *P. anaerobius* induces TLR2/4, which causes the accumulation of reactive oxygen species (ROS) and promotes tumorigenic conditions such as cholesterol synthesis and cell proliferation.^55^

Even though *P. stomatis* does not promote CRC, it might induce tumor microenvironment (TME) conditions such as hypoxia. *P. stomatis* ferments carbohydrates to produce saccharides including acetic, isobutyric, isovaleric, and isocaproic acids. In CRC fecal samples, RT-qPCR data showed that *P. stomatis* was abundantly present in consensus molecular subtype-1 of CRC.^54^

*Prevotella intermedia* (*P. intermedia*) is a gram-negative bacterium mainly involved in periodontitis and several inflammatory diseases, as well as in some types of cancers. Studies have shown the association of *P. intermedia* with CRC development in African-American cohorts. Further, *P. intermedia* was present in the multicohort study of 526 metagenomic CRC patents’ fecal samples.^56,57^

As with *P. intermedia*, *Parvimonas micra* (*P. micra*) was abundantly found in CRC patients’ fecal samples, suggesting its role in CRC.^58^ *Parvimonas micra* has been shown to interrupt nucleotide-binding oligomerization domain 2 (NOD2) - an innate immune sensor - leading to the development of periodontitis. Nucleotide-binding oligomerization domain 2 is also involved in cancer progression and chemotherapy resistance, which further suggests that *P. micra* regulation of NOD2 might induce CRC.^59^

Enterotoxigenic *Bacteroides fragilis* (ETBF) is a toxic substance produced by *Bacteroides fragilis* (*B. fragilis*) and is associated with the occurrence of CRC.^60^ In addition, studies have shown that *B. fragilis* is abundant in sporadic and familial adenomatous polyposis CRC. An individual having a high quantity of *B. fragilis* is at risk of developing CRC.^61^ Additionally, tumor-prone animals infected with enterotoxigenic *B. fragilis* and *Escherichia coli* (*E. coli*; producing colibactin) exhibited modification of the pro-inflammatory cytokines IL-17 through activation of (Signal Transducer And Activator Of Transcription 3 [STAT3]) and NF-kB.^62^ Furthermore, the *B. fragilis* toxin (BFT) can damage E-cadherin that causes alterations in cell morphology, and promotes carcinogenesis and irreversible DNA damage.^63^ Furthermore, studies have shown that ETBF regulates cyclooxygenase-2, which leads to upregulation of PGE2 and promotion of inflammation and cell proliferation.^63^ However, nontoxigenic *B. fragilis* induces protective effect and suppresses pro-inflammatory elements to protect from carcinogenic proliferation.^63^

*Streptococcus gallolyticus* (*S. gallolyticus*) plays a role in promoting TMEs as it generates an immunosuppressive microenvironment.^64^ Besides, *S. gallolyticus*-infected patients have elevated mRNA expression of pro-inflammatory markers such as NF-κB and IL-8.^59^ Therefore, further investigation is needed of the *S. gallolyticus* subspecies involved in CRC.

*Escherichia coli* is active in multiple gastrointestinal (GIT) diseases, including IBD and CRC, as it invades the colonic mucosa and becomes intracellular.^65^ In CRC patients, *E. Coli*, which produces a genotoxin colibactin called pks+, is dominant and is involved in DNA double-strand breaks in vivo and in vitro.^66^ *Escherichia coli* pks+ levels are higher in late stages of CRC and tumor tissues than in early stages and adjacent non-tumor tissues.^67^ Other genotoxins produced by *E. coli* are the cyclomodulins cycle inhibiting factor, which is responsible for independently interrupting mitosis than the DNA damage effect.^68^ Moreover, cytotoxic necrotizing factor-1 has been shown to induce chromosomal appearance and genomic instability by imitating the actin cytoskeleton, which leads to senescence.^69^ Computational proteome-wide study predictions of *E. coli* involvement in CRC have shown that *E. Coli* induces CRC by targeting proteins in endoplasmic reticulums, Golgi apparatuses, peroxisomes, nuclei, and mitochondria.^70^

## Bacterial metabolites and CRC

Short-chain fatty acids are one of the gut microbiota products that function to ferment carbohydrates. They regulate the host’s gut microbiota intestinal homeostasis by interacting with G protein coupled receptors (GPCRs).^71^ For example, SCFAs cooperate with GPR43 to modulate T-cells and inhibit histone deacetylation.^72^ In CRC, SCFAs such as butyrate and acetate have been shown to regulate downstream targets of the aryl hydrocarbon receptor, which involves modulating inflammation in the GIT, thereby suggesting a protective synergistic effect against CRC in human cell lines and mouse models.^73^ In addition, treating CRC with butyrate results in rebalance of composition and diversity of gut microbiota.^74^ Therefore, probiotic treatment with *Butyricicoccus pullicaecorum* and *Faecalibacterium prausnitzii* might be used to treat CRC.^75,76^ In addition, acetate - a SCFA product - has been shown to inhibit inflammation and induce apoptosis in CRC cells by activating caspase-3.^77^

Another SCFA product with antitumor activity achieved through modulation of Tc17 cells and CD8+ T cells (or CTLs) is propionate.^78^ Butyrate has been shown to produce anti-inflammatory effects by stimulating T cells and increasing production of the FOXP3 transcription factor.^79^ In addition, in Caco-2, propionate has been found to induce apoptosis by inducing ROS activity and decreasing glucose oxidation.^80^ Furthermore, propionate can inhibit the NF-kB pathway, resulting in suppression of IL-6 production in colon cells.^79^

Bile acid is a metabolite of the gut microbiota that is divided into primary and secondary bile acids. Secondary bile acid increases in response to a high-fat diet, which has been shown to be involved in the development of CRC. Bile acid can interact with a receptor such as the farnesoid X receptor (FXR), which is known for its inhibitory role in cancer development. farnesoid X receptor has been shown to be able to inhibit CRC development by maintaining bile acid homeostasis.^81^ In contrast, high bile acid concentration is associated with downregulation of FXR, resulting in a pro-tumorigenic phenotype.^82^

Several studies have shown that secondary bile acid is a regulator of pro-inflammatory interleukins involved in tumor metastasis and progression. For example, (lithocholic acid) has been shown to promote cancer development by upregulating IL-8, ERK1/2, and inhibiting STAT3 phosphorylation.^83^ The role of secondary bile acid in activating the proliferative pathway has been further demonstrated by examining the mitogen-activated protein kinases (MAPK) pathway. Both the secondary bile acid products LCA and (deoxycholic acid) have been shown to promote CRC progression by activating the MAPK pathway through upregulation of urokinase-type plasminogen activator receptors and calcium signaling.^84^ In addition, DCA has been shown to induce cancer via multiple processes, such as through the stimulation of ERK1/2, poly-ADP-ribose polymerase, and caspase-3 signaling. As a result, mechanisms such as DNA damage, ROS generation, and disruption of retinoblastoma protein levels cause cancer progression.^84^

Lactate is a metabolite produced by gut microbiota and has an essential role in controlling angiogenic effect and TME.^85^ Lactate has been shown to stimulate TME by creating an acidification environment, stimulating angiogenic responses to oxygen transfer, glucose delivery, and nutrition delivery, thereby promoting CRC invasion, proliferation, and migration.^86^

Succinate is another metabolite produced by gut microbiota’s fermentation of fiber. It has a role in both cancer progression and prevention. It has been shown to not only inhibit CRC proliferation but also induce CRC metastasis via succinate receptor signaling. Therefore, investigation of the role of succinate in CRC requires further examination.^87^

Gut microbiota produces several metabolites from the fermentation of protein to amino acids. Among them, hydrogen sulfide has been demonstrated to inhibit colon cancer growth and metatstases through CD44.^88^ Other metabolites produced from a meat-based protein diet are N-nitroso compounds (NOCs). In CRC, NOCs have been shown to contribute to K-ras mutation, which drives CRC proliferation.^89^

## Drug metabolism and absorption

Gut microbiota can influence the metabolism of chemical compounds such as drugs and xenobiotics. Microbiomes can reduce hydrolyzation, decarboxylation, dehydroxylation, dealkylation, dehalogenation, and deamination of endogenous diet compounds and chemical compounds.^90^ For example, gut microbiota mediates a range of IBD treatments such as 5-aminosalicylic acid prodrugs.^91^ Furthermore, gut microbiota manipulates xenobiotic metabolism, which leads to chemicals changing to either inactive or bioactive substances in the small intestine.^92^ For example, in Parkinson’s treatment, Levodopa, is interrupted by *Enterococcus faecalis* and *Eggerthella lenta*.^92^ It is initially decarboxylated by a tyrosine decarboxylase into dopamine and then dehydroxylated into m-tyramine by a dopamine dehydroxylase.^92^ In addition, the efficiency of xenobiotic absorption is related to the composition of gut microbiota that can compete with host metabolites.^93^ For instance, p-cresol (a bacterial metabolite) can compete with the host to conjugate sulfate, which might disturb the absorption of drugs that need to conjugate with the sulfate to function.^90^ In addition, the gut microbiota can affect the production of several enzymes in the host’s small intestine and liver. For example, in germ-free and conventional (CV) mice, the expression of CYP450 (an oxidation regulator gene) is elevated in CV mice, suggesting that microbiota engages in such regulation.^94^

## Gut microbiota and CRC treatment

Metabolites from the gut microbiota have a variety of roles in governing CRC therapy. Propionate is one SCFA metabolite that has been demonstrated to increase HECT domain E3 ubiquitin protein ligase 2, which in turn degrades euchromatic histone lysine N-methyltransferase 2 and inhibits the growth of CRCs.^95^ This promotes tumor necrosis factor-induced protein 1 and results in the downregulation of H3K9me2 metylation, which leads to CRC cell death.^95^ In addition, gut microbiota such as *F. nucleatum*, can be targeted with antibiotics to induce a better response to fluorouraci (5FU) in CRC patients. A study in orthotopic mice showed that the combination of metronidazole and 5FU in nanoparticles to form anti-CRC gel increased treatment efficiency.^96^ Furthermore, targeting *F. nucleatum* was found to be beneficial for restoring tumor-immune microenvironments through a phage-based bioinorganic hybridization system. The M13 filamentous phages were screened to identify an *F. nucleatum*-specific binding phage. Thereafter, silver nanoparticlewere electrostatically bound to the *F. nucleatum*-specific phage, resulting in the destruction of *F. nucleatum* and blocking of the recruitment of immunosuppressive cells.^97^

In conclusion, the role of the gut microbiota in health and disease has been the subject of scientific study for over a decade. Gut microbiota metabolite treatment has also been found to be helpful in reforming gut microbiota composition and preventing cancer. In addition, gut microbiota plays a significant role in the absorption of drugs provided in CRC treatment, which increases treatment efficiency. However, no significant conclusion has been drawn regarding the role of gut microbiota in CRC treatment. Therefore, additional research is needed in this area.
